# Nutrition in CrossFit® – scientific evidence and practical perspectives: a systematic scoping review

**DOI:** 10.1080/15502783.2025.2509674

**Published:** 2025-06-05

**Authors:** Diogo V. Martinho, André Rebelo, Filipe Manuel Clemente, Renato Costa, Élvio R. Gouveia, Adam Field, Juliano Casonatto, Daniel van den Hoek, Krzysztof Durkalec-Michalsk, Michael J. Ormsbee, Hugo Sarmento

**Affiliations:** a University of Coimbra, Faculty of Sport Sciences and Physical Education, Coimbra, Portugal; b Laboratory of Robotics and Engineering Systems, Interactive Technologies Institute, Funchal, Portugal; c Universidade Lusófona, CIDEFES, Centro de Investigação em Desporto, Educação Física e Exercício e Saúde, Lisboa, Portugal; d COD, Center of Sports Optimization, Sporting Clube de Portugal, Lisboa, Portugal; e Instituto Politécnico de Viana do Castelo, Rua Escola Industrial e Comercial de Nun’Álvares, Escola Superior Desporto e Lazer, Viana do Castelo, Portugal; f Sport Physical Activity and Health Research & Innovation Center, Viana do Castelo, Portugal; g Gdansk University of Physical Education and Sport, Gdańsk, Poland; h University of Madeira, Department of Physical Education and Sport, Funchal, Portugal; i Manchester Metropolitan University, Department of Sport and Exercise Science, Institute of Sport, Manchester, UK; j University of North Paraná, Research Group in Physiology and Physical Activity, Londrina, Brazil; k University of the Sunshine Coast, School of Health, Petrie, Queensland, Australia; l Poznan University of Physical Education, Department of Sports Dietetics, Poznan, Poland; m Charles University, Sport Sciences–Biomedical Department, Prague, Czechia; n Florida State University, Department of Health, Nutrition, and Food Sciences, Tallahassee, FL, USA; o Florida State University, Institute of Sports Sciences and Medicine, Tallahassee, FL, USA; p University of KwaZulu-Natal, Discipline of Biokinetics, Exercise, and Leisure Sciences, Durban, South Africa; q University of Arkansas for Medical Sciences, Center for Aging and Longevity, Geriatrics, Little Rock, AR, USA; r University of Coimbra, CIPER, FCDEFUC, Coimbra, Portugal

**Keywords:** Carbohydrates, caffeine, energy, performance, strength, conditioning

## Abstract

**Background:**

CrossFit^Ⓡ^ sessions and competitions are characterized by high-intensity challenges that combine aerobic and anaerobic activities with short recovery periods. As a result, effective nutritional practices play a crucial role in optimizing performance and enhancing recovery. Therefore, nutritional practices are central to optimizing performance and accelerating recovery. This review aims to summarize current evidence on nutritional and ergogenic aid responses to CrossFit^Ⓡ^ practice.

**Methods:**

The search was conducted in four electronic databases (PubMed, Web of Science, Scopus and SportDiscus). Gray literature was also extracted for studies exploring the nutritional habits of CrossFit^Ⓡ^ participants as well as the ingestion of ergogenic aids. In addition, a meta-analysis was conducted to examine the impacts of dietary habits and ergogenic aids on performance.

**Results:**

Forty-nine studies met the eligibility criteria and were included in the current review. Carbohydrate intake was below the recommendations for athletes, although protein ingestion remains adequate. High-carbohydrate diets had a positive effect on CrossFit^Ⓡ^ performance. The evidence concerning the effects of a ketogenic diet on performance is limited. When used prior to or during the workout, the impact of carbohydrates on CrossFit^Ⓡ^ performance was negligible, whereas the effect of caffeine was significant. Ergogenic aids, particularly creatine and protein, are commonly used by CrossFit^Ⓡ^ participants.

**Conclusion:**

The standard diets recommended to CrossFit^Ⓡ^ participants need to be revised because they are characterized by lower values of carbohydrates. Caffeine should be used prior to or during the CrossFit^Ⓡ^ sessions. Regarding the impact of ergogenic aids on recovery, future studies are needed.

## 1. Background

CrossFit^Ⓡ^ has gained worldwide popularity over the last decade. According to data from the World Metrics Report [1], the number of CrossFit^Ⓡ^ affiliations increased by 118% from 2005 to 2015. The CrossFit^Ⓡ^ Open, an event that allows competitors to participate for three weeks, included 344,396 participants in 2024. In CrossFit^Ⓡ^, athletes are required to train distinct components of fitness (cardiorespiratory, stamina, strength, flexibility, power, speed, coordination, agility, balance, and accuracy) and a variety of movements (e.g. deadlift, squat, power clean, push-ups, pull-ups, burpees, rowing, running, cycling) at high intensities and with short periods of recovery [2,3]. Optimizing performance and recovery are two central aspects of success in CrossFit^Ⓡ^ participation [4–6]. Many studies have described the physical and physiological aspects of CrossFit^Ⓡ^ workouts [4,7,8] and have focused on examining the time needed to recover from a specific workout [9–11]. While characterizing CrossFit in terms of physical and physiological demands based on a single workout requires caution, data from Spanish CrossFitⓇ participants indicate that restoring normal levels of physical performance and creatine kinase typically takes 48 to 72 hours [12]. Among Brazilian practitioners, 48 hours of recovery allowed them to reestablish physical performance, whereas creatine kinase values were higher 72 hours after the CrossFit^Ⓡ^ training than before the workout [9]. Therefore, adequate nutritional practices and appropriate nutritional ergogenic aids can optimize performance and accelerate recovery [13,14].

The dietary recommendations for CrossFit^Ⓡ^ participants relate to following the Paleo-or Zone Diets [15]. The Paleo Diet emphasizes the consumption of unprocessed foods, while the Zone Diet focuses on maintaining stable glucose levels through a macronutrient distribution of 40% carbohydrates (CHO), 30% protein, and 30% fat. Both dietary practices tend to neglect the importance of CHO [16,17], which is surprising given the wealth of evidence pertaining to the impact of ingesting CHO before, during, and after training to improve performance and reduce fatigue [18–20]. Two recent reviews published in 2021, including 14 and 13 studies, summarized the effects of dietary and supplementary interventions among CrossFit^Ⓡ^ participants with a focus on performance [21,22]. However, assessments of energy and macronutrient intake among CrossFit^Ⓡ^ participants are limited. This gap needs particular attention to determine whether CrossFit^Ⓡ^ participants meet the nutritional recommendations established for athletes. Additionally, neither of the reviews addressed the prevalence of supplements used in CrossFit^Ⓡ^. Given the rising popularity of CrossFit^Ⓡ^, an increase in the number of studies in the field of nutrition over the past few years is expected. Therefore, the available information regarding nutritional issues in CrossFit^Ⓡ^ needs to be systematically reviewed and summarized.

To improve the dietary practices of nutritionists working with CrossFit^Ⓡ^ athletes, this systematic scoping review aims to do the following: (1) summarize the dietary practices of participants, (2) describe nutritional and energetic intake, (3) examine the acute and chronic effects of nutritional ergogenic aids on performance and recovery, and (4) identify the gaps in the available literature and provide suggestions for future research.

## 2. Methods

The present scoping review conformed to the standards set by the latest revision of the Cochrane guidelines [23] and followed the written instructions proposed by the PRISMA 2020 guidelines [24] as well as the respective extension for scoping reviews [25]. The protocol was also preregistered on the INPLASY plataform (doi:https://doi.org/10.37766/inplasy2024.6.0059).

### 2.1. Eligibility criteria

Original manuscripts published in peer-reviewed journals, master dissertations, doctoral theses, research reports, doctoral dissertations, conference presentations, abstracts, and clinical trials written in English, Portuguese, and Spanish were included in the present review. The Participants, Intervention, Comparator, Outcomes, and Study Design (PICOS) framework was used to define the eligible studies for the present review: Participants – adult CrossFit^Ⓡ^ participants; Intervention – studies that described dietary practices or examined the effects of nutrition on performance or recovery; Comparator – studies assessing the impact of nutrition on performance or recovery versus a placebo or control; Outcomes – energy and/or macronutrient intake, energy expenditure, energy balance, performance, and/or recovery; Study design – observational and interventional studies. No restrictions were applied in terms of publication date.

### 2.2. Search information and information sources

The search strategy included the combination of the following terms: ((nutrition* OR “*nutritional strategy*” OR “*nutritional intervention*” OR diet*, *carbohydrate* OR *glucose* OR *protein* OR *collagen* OR *fat* OR ketone* OR antioxidant* OR *vitamin* OR polyphenol* OR *fruit* OR *creatine* OR *caffeine* OR nitrate* OR *beetroot* OR “*tart cherry*” OR *beta alanine* OR *sodium bicarbonate* OR supplement*OR energy* OR macronutrient* OR micronutrient* OR mineral* OR electrolyte*) AND *CrossFit^Ⓡ^*). The search was divided into two different phases: 1) identification of studies via databases and 2) identification of studies via other methods (dissertation and thesis databases; gray literature databases; trial results in platforms; other systematic reviews; and reference lists of included studies). Four electronic databases were consulted: PubMed, Web of Science, Scopus and SportDiscus. The full search strategy for each database can be found in Supplementary Material 1. The Open Access Thesis and Dissertations database (https://otad.org) was used to check search for master dissertations and doctoral theses. Following the Cochrane guidelines, two platforms were used to identify trial registers (https://clinicaltrials.gov/; https://trialsearch.who.int/) [26]. The references of other systematic reviews on the same topic were consulted in the Web of Science database to identify other potential references. The search strategy adopted was similar to that mentioned above, including the Boolean connector “AND” with the term “systematic review.” The titles of the reference lists of those studies included in the present review were consulted to identify additional studies. The search strategy was conducted on the same day for all databases and platforms (11 June 2024).

Dedicated computer software was used for reference management, facilitating deduplication and screening steps (EndNoteTM 21.0, ClarivateTM). Following the automatic omission of duplicates, two authors manually screened the remaining references for their relevance (DVM/HS). The titles and abstracts were screened first. The full texts of the studies were then screened to ensure that the studies met the eligibility criteria. Two researchers (DVM/HS) conducted screenings, and when necessary, a third author (AR) was contacted to resolve any disagreements.

### 2.3. Data extraction and items

The first author (DVM) created a template to organize the relevant data. The Microsoft Excel^Ⓡ^ document included four sheets: (1) prevalence of nutrition use, (2) data concerning dietary and nutritional intake, (3) the effects of dietary interventions on performance, and (4) the effects of nutrition on performance and recovery. To examine the prevalence of nutritional aids, the following information was retrieved: sample size, percentage of nutritional ergogenic aid use, and percentage consumed by athletes. Data related to energetic and nutritional intake included the relative and absolute values of daily energy expenditure, intake, and macronutrients. Information surrounding the effects of specific diets on performance considered the study design, sample size, intervention, performance outputs, specificity of the performance variable (i.e. CrossFit^Ⓡ^ movements or functional capacities), and mean and standard deviation of both conditions. Corresponding information was collected for studies that tested the effects of nutritional aids on performance and recovery. The corresponding authors were individually contacted when the data were not reported. When the data were presented graphically, specific software was used to extract the data (http://www.getdata-graph-digitizer.com). This software has been shown to be accurate and precise in extracting mean and standard deviation values from graphs [27].

### 2.4. Statistical analysis

#### 2.4.1. Energy and nutritional intake

Sex, sample size, and the means and standard deviations of energy and macronutrient intake were retrieved from each study. The overall means of energy, CHO, and protein contents were split by sex. In parallel, data were organized according to the year of study publication to estimate the tendency of energetic, CHO, and protein intake across time using the moving averages.

#### 2.4.2. Percentage of use of nutritional ergogenic aids

Supplementation prevalence was examined based on sample size and percentage of supplements used by CrossFit^Ⓡ^ participants. The number of cases was calculated based on the preceding information, and a random effect model was considered. The mean prevalence, 95% confidence intervals, and I^2^ were retrieved for the analysis. The I^2^ reflects the proportion of true variance to observed variance, contrasting the true and observed effects.

#### 2.4.3. Interventions (diets and ergogenic aids)

Standardized mean differences corrected by the degrees of freedom and expressed as effect size (i.e. *Hedges’s g*) were used to compare CrossFit^Ⓡ^ performance after the implementation of diets with higher values of CHO or specific acute nutritional strategies (CHOs and caffeine). The effect size calculation was based on a random effects model. The effect sizes were interpreted as follows: < 0.2, trivial; 0.2–0.6, small; > 0.6–1.2, moderate; > 1.2–2.0; large; > 2.0–4.0, very large; and > 4.0, extremely large [28]. An integrative approach was chosen to combine multiple effect sizes of the same study because they represent different features of CrossFit^Ⓡ^ performance [29]. Heterogeneity was assessed via the I^2^ and qualitatively described as follows: low (I^2^ <25%), moderate (25–75%), and > high (I^2^ >75%) [30]. The I^2^ and 95% confidence intervals were used to investigate heterogeneity. The bias of publication was graphically inspected with a funnel plot and statistically verified with Egger’s test (observed Hedge’s g values were contrasted with the respective standard errors). The trim-and-fill method of Duval and Tweedie was used to adjust for potential publication biases [31].

The meta-analysis was performed via Comprehensive Meta-Analysis software version 2.2.064 (BiostatTM, Englewood, NJ, USA).

#### 2.4.4. Risk of bias

The risk of bias was evaluated via two different tools according to the study design: (1) the Quality Assessment Tool for Observational Cohort and Cross-Sectional Studies [32] and (2) the PEDro scale, an 11-item validated tool used to measure the risk of bias and statistical reporting of clinical trials (https://pedro.org.au/english/resources/pedro-scale/). The first tool included fourteen items about the research question, study population, groups recruited from the same population and uniform eligibility criteria, sample size justification, exposure assessed prior to measurement outcome, sufficient timeframe to observe an effect, different levels of the exposure effect, exposure measurement, repeated exposure assessment, outcome measurement, blinding of outcome assessors, follow-up rate and statistical analysis. Each item was analyzed individually and assigned a designation of yes, “no,” or ‘not reported or applicable. The 11-item PEDro scale presents questions about the eligibility criteria, group allocation, group similarities at baseline, blinding procedures, completion rates of the outcome measures, statistical analyses, and reporting of outcome measures. Each item was assigned a value of “yes” (corresponding to 1 point) or “no” (corresponding to 0 points). The first item is not used to calculate the PEDro score. The methodological quality of the interventional studies was interpreted using the following criteria [33]: 0–3 points was considered “poor” quality, 4–5 points was considered “fair” quality, 6–8 points was considered “good” quality, and 9–10 points was considered “excellent” quality. Two authors (DVM and AR) independently assessed the risk of bias. In the event of disagreements, a third author (HS) was consulted, and a final decision was reached by consensus. No studies were excluded based on their assessed risk of bias.

## 3. Results

### 3.1. Study identification and selection

**Figure 1.**
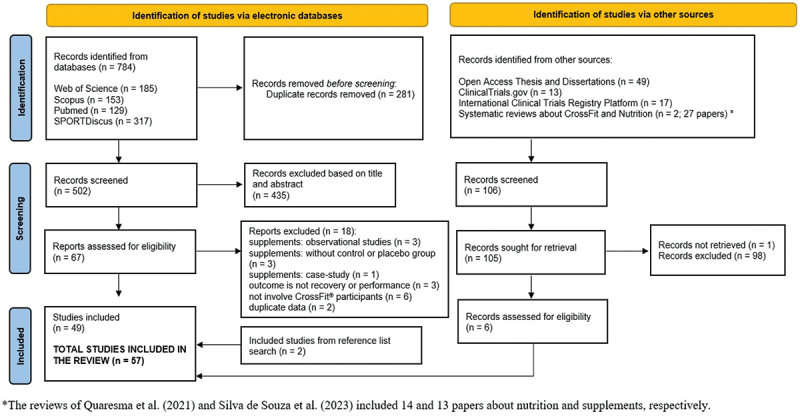
Flow chart of records identification, screening and inclusion in the present review articleCHO (carbohydrates).

The search was conducted in four databases, and 784 records were identified. Of these, 281 were removed because they were identified as duplicates. Thus, the titles and abstracts of 502 articles were screened. After this process, 67 manuscripts remained potentially eligible for the present review. Six reasons were identified to exclude 18 reports: observational studies of supplement intake (*n* = 3) that did not include control or placebo groups (*n* = 3), one case study (*n* = 1), outcomes were not recovery or performance (*n* = 3), the sample was not mentioned as CrossFit^Ⓡ^ participants or athletes (*n* = 6), and duplicate data (*n* = 2). Duplicate data refer to records extracted from the same original papers and abstracts published at conferences. Forty-nine papers extracted from the four databases met the inclusion criteria and were included in the present review. The reference lists of the 49 papers were individually reviewed, and two additional references were considered for the current review. Six master’s or doctoral theses were identified via the Open Access Thesis and Dissertations database. One master’s thesis was not publicly available (https://digitalcommons.lib.uconn.edu/gs_theses/134/). The first author (DVM) contacted the author of the thesis, and it was confirmed that access to the PDF document was unavailable. Consequently, this record was not retrieved for the present review. Another record was found in the International Clinical Trials Registry Platform of the World Health Organization. Six records were identified via other sources and were combined with 51 original papers. The present review included 57 records, as shown in Figure 1.

### 3.2. Study characteristics

Table 1 summarizes the primary information extracted from each study about the use of ergogenic aids and dietary and nutritional patterns of CrossFit^Ⓡ^ participants (e.g. sample size, origin of sample, objective, methods used, and main results) [34–56]. Studies were conducted mainly on Brazilian participants (*n* = 16, ~70%), and four included American participants (~17%). The total number of CrossFit^Ⓡ^ participants in this group of studies was 4,560. Two main topics emerged from Table 1: (1) food, energy, and nutritional intake [34,35,37,40–45,47–52,54,55] and (2) the use of supplements [36,38–40,43,44,46,50,52,53,56] The percentage and type of supplements used were evaluated via questionnaires, whereas the daily energy and nutritional intake were estimated via 24-hour recall methods or food diaries.

**Table 1.** Studies about ergogenic aids statistics, energetic, and nutritional characteristics of CrossFit® participants.

Study	Sample characteristics	Country	Methodology – nutritional/ergogenic aids issues	Main results
De Jesus et al. [34]	4 high performance male and female athletes (19–30 yrs); training experience: NR.	Brazil	24 hours recall	– Nutritional intervention ↑ energy and macronutrients intake.
Brustolin et al. [35]	20 CrossFit® male and female participants (28.7 ± 4.5 yrs); training experience: NR.	Brazil	Interview	− 40% performed 3–4 meals per day. – Fruits, vegetables, cereals, legumes and milk and dairy products are ↑ selected. − 25% used supplements. − 75% ingested a pre-workout 30 minutes prior to the CrossFit® section: banana, oats, cinnamon and honey. − 70% reported that ingested 1 hour post CrossFit® workout: different foods.
Dos Santos et al. [36]	112 CrossFit® male and female participants of different competitive levels (28.9 ± 7.6 yrs); training experience: > 1 yr.	Brazil	Questionnaire	− 78% of participants used supplements. – Supplements were mainly used to ↑ performance. – >50% of participants used supplements reported to use supplements at least 5 or more times per week. – Protein and creatine were used by 63% and 48% of participants, respectively.
Rezende et al. [37]	20 male and female CrossFit® participants; training experience: NR.	Brazil	24 hours recall	– ↔ energy intake and relative protein between groups. – ↓% CHO and ↑ fat in CrossFit® participants.
Faria et al. [38]	113 CrossFit® male and female participants; training experience: < 3 mths to >2 yrs.	Brazil	Questionnaire	− 75% of participants used supplements. − 81% of participants used whey protein (↑ recovery and muscle mass). − 53% of participants used creatine (↑ performance and muscle mass). − 53% of participants used BCAAs (↑ recovery).
Comerlatto et al. [39]	217 male and female CrossFit® participants (33.7 ± 5.2 yrs); training experience: NR.	Brazil	Questionnaire	− 61% of participants used supplements. − 53% ingested whey protein. − 31% ingested creatine.
Filho et al. [40]	160 male and female CrossFit® participants (25–30 yrs); training experience: NR.	Brazil	Questionnaire: supplementation; intake; food pattern: food frequency questionnaire	− 98% of participants used supplements. – Creatine was ingested by 61% of participants. – Why protein was ingested by 28% of participants. – ↓ number of participants used supplements prior or post training. – Fruits, legumes, milk and dairy products are ↑ selected.
Pearson and Jenkins [41]	443 CrossFit® male and female participants (36.6 ± 11.4 yrs); training experience: > 6 mths.	US	Dietary health questionnaire	– Dietary intake was associated weight status, sex, age, exercise and nutritional goals.
Gonçalves et al. [42]	25 CrossFit® male and female athletes (32.0 ± 8.9 yrs); training experience: NR.	Brazil	Food diary	– The mean energy intake was 2844 ± 724 kcal.day^−1^. – The relative intake of CHO and protein were 4.6 g.kg^−1.^day^−1^ and 2.2 g.kg^−1.^day^−1^, respectively. − 64% of athletes consumed ↓ CHO than the recommendations. – ↓ values of micronutrients (calcium, potassium, selenium, vitamins A, B9, B12, and D) ingestion were also noted.
Mesquita and Cavalcanti [43]	24 CrossFit® male and female participants (33.6 yrs); training experience: NR.	Brazil	Food frequency questionnaire	– ↑ salad, vegetables, roots, fruits, legumes, rice or pasta, milk and dietary products, meat, fish and eggs. − 75% of the food ingested was derived from protein sources. – ↓ of participants used supplements (21%).
Brisebois et al. [44]	2576 CrossFit® male and female participants (33.6 yrs); training experience: NR.	US,Canada, UK, Australia	Questionnaire	− 60% of participants practiced a specific diet. − 82% use supplements twice per week. – Macro Counting, intermittent fasting, paleo diets were ↑ reported. − 2015 supplements were described. – Protein was the most used supplement (51%), followed by creatine (23%) and pre-workouts (21%). – Nutrition was recognized as determinant for CrossFit® performance. – CHO and protein were widely recognized as determinant for performance.
Vieira et al. [45]	12 male recreational CrossFit® participants (30.2 ± 5.5 yrs); training experience: NR.	Brazil	Food diary	– The mean energy intake was 2561 ± 593 kcal.day^−1^. – The relative intakes of CHO and protein were 3.5 ± 1.2 g.kg^−1.^day^−1^ and 1.5 ± 0.5 g.kg^−1.^day^−1^, respectively. – Fat represents 34% of total daily energy intake.
Higino and Freitas [46]	450 male and female CrossFit® participants; training experience: < 3 mths to >2 yrs.	Brazil	Questionnaire	− 71% of participants used supplements. − 31% of participants ingested protein supplements.
Kutch [47]	73 female CrossFit® participants; training experience: 3.3 yrs.	US	Food diary	– Most of CrossFit participants did not meet the nutritional athletic recommendations. − 30% were ↓ 30 kcal.kgFFM^−1^.day^−1^.
Gogojewicz et al. [48]	62 male and female CrossFit® participants; training experience: > 6 mths.	Poland	Food diary	– The energy intake and expenditure of male participants were 2265 ± 417 kcal.day^−1^ and 2828 ± 316 kcal.day^−1^. – Corresponding values for females were 1736 ± 407 kcal.day^−1^ and 2598 ± 286 kcal.day^−1^. – The protein values were similar in male and female groups (1.6 g.kg^−1.^day^−1^). – Females ingested 3.9 g.kg^−1.^day^−1^ of CHO whilst males ingested 3.3 g.kg^−1.^day^−1^. – The % of fat intake was 30% in both groups. – Among females, ↓ calcium, iron, zinc, acid folic and ↑ phosphorus, magnesium.
Zaykova [49]	25 male and female professional CrossFit® participants; training experience: 3.5 yrs.	Bulgaria	Questionnaire	– Daily energy intake ↑ than daily energy expenditure. – Only five participants ingested >2.0 g.kg^−1.^day^−1^ of protein.
Brescansin et al. [50]	30 CrossFit® male and female participants (29.4 ± 9.1 yrs); training experience: NR.	Brazil	Food frequency questionnaire	− 33.3% of participants use supplements. – Milk and diary products, eggs, legumes, cereals, fruits were consumed at least once a week.
Terry [51]	21 male and female CrossFit® participants; training experience: NR.	US	Food frequency questionnaire	– Female CrossFit® participants had ↓ calcium intake. – Male CrossFit® participants had ↓ calcium and magnesium intake.
Fayad [52]	15 male and female CrossFit® athletes (25–41 yrs); training experience: NR.	Brazil	24 hours recall	− 60% of athletes ingested supplements. – Whey protein and creatine were used by 53% and 26% of athletes, respectively. – Mean energy intake was 1739 kcal.day^−1^ (range: 835–1739 kcal.day^−1^). – Relative intake of CHO was 2.3 g.kg^−1.^day^−1^. − 67% of athletes ingested >1.7 g.kg^−1.^day^−1^ of protein.
Lins et al. [53]	50 male and female CrossFit® participants (30.2 ± 5.6 yrs); training experience: < 1 mth to >1 yr.	Brazil	Questionnaire	− 80% of participants ingested supplements. – All participants that ingested supplements used whey protein, creatine, glutamine and BCAAs.
Pacheco et al. [54]	10 male and female CrossFit® participants (27.9 ± 7.4 yrs); training experience: NR.	Portugal	Food frequency questionnaire	– CrossFit® participants tend to consume ↓ dairy products, vegetables, legumes and sweets and pastries.
Bueno et al. [55]	10 male CrossFit® participants (18–50 yrs); training experience: > 6 months.	Brazil	Food diary	– The median energy intake 1409 kcal.day^−1^. – The median relative intake of protein, CHO and fats were 4.0 g.kg^−1.^day^−1^, 11.5 g.kg^−1.^day^−1^, 2.0 g.kg^−1.^day^−1^, respectively. – Micronutrients (vitamins C, E, K) were ↓ the recommendations whilst B6 and B12 were ↑. – Calcium and magnesium were ↓ the recommendations and iron and zinc ↑.
Freitas et al. [56]	88 male and female CrossFit® athletes; training experience: < 1 yr to >3 yrs.	Portugal	Questionnaire	− 76.1% of CrossFit® athletes ingested at least one supplement. – Protein, creatine, and BCAAs are ↑ by CrossFit® athletes.

Table 2 presents the studies that focus on investigating the effects of a specific type of diet on physical performance [57–63]. The sample sizes of these studies ranged from 11 to 27 male and female CrossFit^Ⓡ^ participants. Three studies tested the effects of diets with a considerable percentage of carbohydrates [58,59,62], three papers focused on the impact of fat (i.e. a ketogenic diet) [60,61,63], and one study investigated the influence of fasting on performance [57]. The time of interventions and performance outputs varied across studies.

**Table 2.** Studies that examined the effects of dietary interventions in CrossFit® participants.

Study	Sample characteristics	Study design	Diet			Main results
			Intervention	Control	Time	
Eroglu et al. [57]	11 female CrossFit® participants (30.9 ± 3.4 yrs); training experience: > 2 yrs.	Crossover	Fasting: consumed only water	Non-fasting: meal prior 2–3 hours before the exercise (50% CHO, 25% fats, 25% protein)	24 hours	– Blood lactate ↑ eating trial at baseline but, at post-exercise was ↑ fasting trial. – ↔ RPE, handgrip strength, jumping, CrossFit® performance time. – ↔ Heart rate.
Ficarra et al. [58]	22 male and female CrossFit® participants; training experience: > 1 yr.	Parallel	Mediterranean diet (50% CHO, proteins were calculated based on training section, fats were distributed for the remaining calories)	Control group: habitual diet	8 weeks	– Relative peak power ↑ in both groups. – Maximal speed ↑ in mediterranean diet. – Time to attain peak power ↓ in mediterranean diet. – Power drop ↑ in mediterranean diet. – Jump height and time ↑ in Mediterranean diet. – Fran performance and chin-up test ↑ in Mediterranean diet. – Push-up test ↑ in both groups.
Durkalec-Michalski et al. [59]	20 male and female CrossFit® participants; training experience: > 1 yr.	Parallel	Vegan diet (adjustments of macronutrients – CHO: 395.3 ± 63.1 g.kg^−1^; protein: 113.7 ± 23.4 g.kg^−1^; fat: 62.3 ± 21.2 g.kg^−1^)	Mixed diet (adjustments of macronutrients – CHO: 297.7 ± 53.9 g.kg^−1^; protein: 130.1 ± 23.7 g.kg^−1^; fat: 98.9 ± 12.7 g.kg^−1^)	4 weeks	– Number of repetitions on deadlift and squat ↑ in both groups. – ↔ fight gone bad performance.
Durkalec-Michalski et al. [60]	22 male and female CrossFit® participants; training experience: > 2 yrs.	Crossover	Ketogenic diet (fats: >75%, protein: 1.7 g.kg^−1^, CHO: ≤ 5%)	Customory diet	4 weeks	– ↔ maximal oxygen uptake, time to exhaustion, maximal power, fight gone bad CrossFit® challenge. When the analysis was split by sex: – maximal oxygen uptake ↓ 10% in females that followed a ketogenic diet. – maximal heart rate ↑in females that followed a ketogenic diet. – heart rate gas exchange threshold ↑in males that followed a ketogenic diet.
Kephart et al. [61]	12 male and female CrossFit® participants; training experience: NR.	Parallel	Ketogenic diet	Customary diet	12 weeks	– ↔ maximal repetition of squat and power clean, push-ups, 400-m running in both groups. – Customary diet ↑ maximal oxygen uptake.
Escobar et al. [62]	18 male and female CrossFit® participants; training experience: NR.	Parallel	CHO: 6–8 g.kg^−1.^day^−1^	Lower CHO: < 6 g.kg^−1.^day^−1^	3 days	– CrossFit® performance ↑ CHO diet. ↔ maximal oxygen uptake, blood lactate in both groups.
Gregory et al. [63]	27 male and female CrossFit participants®; training experience: > 1 month.	Parallel	Ketogenic diet (CHO <50 grams per day)	Customary diet	6 weeks	– Ketogenic diet and customary diet ↔ physical performance.

The characteristics of the studies (study design, dosage, timing, and main findings) of ergogenic supplements are shown in Table 3. Sodium bicarbonate [66,67,72,73,79,85] carbohydrates [64,70,86,87], caffeine [65,74,80,84], betaine [69,81], capsiate [71], beta-alanine [75], tribulus terrestris [76,78], nitrate [82,88], and beetroot juice [83] were tested in CrossFit^Ⓡ^ participants. The combination of different supplements was also investigated in four studies [68,77,89,90]. Most of the supplements were ingested before the CrossFit^Ⓡ^ workout [65–68,70,71,73,74,76,79–85,87,89,90], two studies investigated the effects of supplements during the workout [64,87], and one study tested the impact of CHO intake prior to and during the workout [86].

**Table 3.** Ergogenic aids interventions in CrossFit® participants.

Study	Sample characteristics	Study design	Intervention			Main findings
			Supplement	Dosage	Timing	
Grijota et al. [64]	21 male CrossFit® athletes; training experience: > 2 yrs.	Double-blind crossover trial	Cyclodextrin (CHO derived from plant starch).	30 g	During	– ↔ CrossFit® performance, heart rate, rate of perceived exertion, lactate, glucose execution speed in bench press between groups. – ↑ power values in CHO group. – Average power ↑ in CHO group whilst fatigue index ↑ placebo group.
Główka et al. [65]	26 male and female CrossFit® athletes; training experience: > 2 yrs.	Double-blind crossover trial	Caffeine	3 mg.kg_BM_^−1^, 6 mg.kg_BM_^−1^, 9 mg.kg_BM_^−1^	70 minutes prior to the test	– ↔ of CrossFit® performance (number of repetitions in both groups). − 6 mg.kg_BM_^−1^ CAF ↑ number of repetitions. – Heart rate and rate of perceived was ↔ in both groups. – Blood lactate was ↑ in CAF 9 mg.kg_BM_^−1^ in comparison to CAF 3 mg.kg_BM_^−1^. − 6 mg.kg_BM_^−1^ CAF ↓ reaction and motor times.
Durkalec-Michalski et al. [66]	30 male and female CrossFit® participants; training experience: > 4 yrs.	Double-blind crossover trial	Sodium bicarbonate	0.15 g^·^kg _FFM_^−1^, 0.25 g^·^kg _FFM_^−1^. 0. 35 g^·^kg _FFM_^−1^	2 hours prior to exercise	– ↑ blood pH 0.25 g^·^kg _FFM_^−1^ and 0.35 g^·^kg _FFM_^−1^ NaHCO_3_ to the baseline values at recovery. – ↑ blood pH 0.35 g^·^kg _FFM_^−1^ NaHCO_3_ at recovery. – Lymphocytes ↑ 0.25 g^·^kg_FFM_^−1^ NaHCO_3_ post-exercise and recovery than control. – Granulocytes, red blood cells, hemoglobin, white blood cells ↑ control at recovery. – Monocytes ↑ 0.25 g·kg_FFM_^−1^ NaHCO_3_ post-exercise than control at recovery. – Magnesium ↑ in control at recovery. – Creatinine ↓ in control at recovery.
De Souza et al. [67]	17 advanced male CrossFit® trained athletes; training experience: > 1 yr.	Double-blind crossover trial	Sodium bicarbonate	0.30 g^·^kg^−1^ ingested with the meal	2 hours prior to exercise	– NaHCO_3_ ↑ the performance in Fran CrossFit® challenge. – Performance in 500 m rowing and jumping ↔. – NaHCO_3_ ↑ blood pH in comparison to placebo. – No differences between groups were found for lactate, heart rate and RPE.
Ziyaiyan et al. [68]	20 male CrossFit participants®; training experience: > 2 yrs.	Double-blind crossover trial	Caffeine + sodium bicarbonate	6 mg^·^kg^−1^ (CAF) + 0.1 g^·^kg^−1^ (NaHCO_3_)	120, 90, 60 minutes before the protocol	– No significant effects of supplementation were noted. – HR_max_ ↑ CAF and CAF + NaHCO_3_ in comparison to control group. – RPE during Cindy challenge ↓ CAF compared to NaHCO_3_, CAF + NaHCO_3_ and control groups.
Zawieja et al. [69]	43 male recreationally and regularly trained CrossFit® participants; training experience: > 1 yr.	Double-blind crossover trial	Betaine	2.5 g^·^kg^−1^, 5.0 g^·^kg^−1^	3 weeks: 2.5 g^·^kg^−1^ – three capsules in the morning and two in the evening; 5.0 g^·^kg^−1^ – four capsules in the morning, three in the afternoon, and three in the evening	– BET ↑ 8.7% Fight Gone Bad performance but, no significant differences were found compared to placebo group. – ↔ performance in Wingate test in both groups.
Mattos et al. [70]	9 male CrossFit® participants; training experience: > 3 months.	Crossover	CHO	1.0 g^·^kg^−1^ grape + banana juice; 1.0 g^·^kg^−1^ Whey Maximize	60 minutes before the protocol	– RPE was ↓ on Whey Maximize supplement than fast state.
Oliveira et al. [71]	17 male trained CrossFit® participants; training experience: > 1 yr.	Double-blind crossover trial	Capsiate	12 mg, 24 mg	45 minutes before the protocol	– Performance, HR and RPE was ↔in CAP and placebo.
Martin et al. [72]	11 female CrossFit® athletes; training experience: > 2 yrs.	Double-blind crossover trial	Sodium bicarbonate	0.3 g^·^kg^−1^	Individualized approach	– NaHCO_3_ ↑ 2.2% rowing performance in comparison to placebo group. – Mean power ouput ↑ NaHCO_3_ (*p* < 0.05).
Gomes et al. [73]	6 male CrossFit® participants; training experience: > 6 months.	Double-blind parallel trial	Sodium bicarbonate	0.3 g^·^kg^−1^	90 ^–^ 150 minutes prior to the test	– No effects of NaHCO_3_ on RPE.
Caetano et al. [74]	8 male trained CrossFit® participants; training experience: > 2 yrs.	Double-blind crossover trial	Caffeine	6 mg^·^kg^−1^	60 minutes prior to the protocols	– CAF ↑ local muscular endurance and number of repetitions. – Squat maximum repetition was ↔ in both groups.
Silvestre [75]	19 male and female CrossFit® participants; training experience: > 2 months	Double-blind crossover trial	Beta-alanine	6.4 g.day^−1^	28 days. 4 times per day: 3 hours of interval (especially after meals)	– No differences between groups in performance, RPE, sleep quality, mood and well-being.
Fernandez-Lázaro et al. [76]	30 trained male CrossFit® participants; training experience: > 3 yrs.	Parallel single-blind trial	Tribulus terrestris	770 mg	6 weeks: 30 minutes prior to CrossFit® workout	– Muscle biomarkers: ↑ LDH in placebo group whilst LDH ↓ in TT supplementation. – Inflammatory markers: ↑ CRP placebo group whilst CRP ↓ in TT supplementation but without significance. – Total Oxidant Status ↑ in TT group. – Performance ↔ in both groups.
Maroufi et al. [77]	8 male CrossFit® athletes; training experience: > 6 months.	Crossover single-blind trial	Carbohydrates + protein	500 ml carbohydrate-protein with different ratios (2:2, 3:1)	60 minutes prior to the test and immediately before	– Performance in CHO + protein and placebo groups was ↔ considering the number of repetitions in CrossFit® challenges.
Fernandez-Lázaro et al. [78]	30 trained male CrossFit® participants; training experience: > 1.5 yrs.	Parallel single-blind trial	Tribulus terrestris	770 mg	6 weeks	– TT ↑ bench press performance significantly whilst the performance in control group ↔. – CrossFit® performance, RPE, hormonal indicators ↔ in both groups.
Toledo et al. [79]	30 male experienced CrossFit® participants; training experience: 2.2 yrs.	Double-blind crossover trial	Sodium bicarbonate	0.3 g^·^kg^−1^	60 minutes prior to the test	– Number of repetitions, lactate, average and maximal heart rate, were ↔ in both conditions.
Stein et al. [80]	30 male CrossFit® participants; training experience: > 6 months.	Double-blind crossover trial	Caffeine	5 mg.kg^−1^	60 minutes prior to the test	– Number of repetitions and RPE ↔ in both groups.
Moro et al. [81]	29 male and female CrossFit® participants; training experience: > 6 months.	Double-blind crossover trial	Betaine	2.50 g (dosages of 1.25 g)	6 weeks: first dosage in morning, second dosage 60 minute before the work	– Significant improvements on back squat were noted for BET. – No differences between groups were found for the remaining performance variables.
Ricordi et al. [82]	18 male and female beginner CrossFit® participants; training experience: > 6 months.	Double-blind crossover trial	Nitrate	10 g.kg^−1^	2 hours before CrossFit® practice	– CrossFit® performance was ↔ in both groups.
Garnacho-Castano et al. [83]	12 male CrossFit® participants; training experience: > 2 yrs.	Double-blind crossover trial	Beetroot juice	~808 mg	3 hours prior to exercise	– BJ ↑ the number of repetitions (after including resting time in the protocol). – Jumping and blood lactate were ↔in both conditions. – SpO_2_ decrease was ↑ BJ condition. – Decline in jumping performance was ↑ BJ condition.
Fogaça et al. [84]	9 male experienced CrossFit® participants; training experience: 2 yrs.	Double-blind crossover trial	Caffeine	6 mg.kg^−1^	60 hours prior to exercise.	– Negligible differences between CAF and placebo groups were found for propulsive and peak power, bench press, jump squat, countermovement jumps and handgrip strength. – RPE and DOMS were ↔ in both groups. – Concentrations of CK and CPR ↔ in post and 24 hours post workout.
Durkalec-Michalski et al. [85]	21 recreational male and female CrossFit® participants; training experience: > 2 yrs.	Double-blind crossover trial	Sodium bicarbonate	Progressive dosages: 150 mg.kg^−1^. Each 2 days, for 8 days, ↑ 25% of sodium bicarbonate dosage.	10 days: 3 doses per day. Training days was taken 1.5 hours before training sessions.	– Performance in FGB ↑ when participants ingested NaHCO_3_. – Derived from incremental cycling test, maximum workload and HR were ↔ in both groups. – Workload at ventilatory threshold was 4.6% higher (*p* < 0.05) in NaHCO_3_ than placebo. – Time to attain ventilatory threshold was 5.1% higher (*p* < 0.05) in NaHCO_3_ than placebo. – HR at ventilatory threshold was significantly higher in NaHCO_3_ than placebo. – Time to attain ventilatory threshold was 5.1% higher (*p* < 0.05) in NaHCO_3_ than placebo. – Pyruvate was significantly higher in NaHCO_3_ than placebo.
Rountree et al. [86]	8 CrossFit® trained males; training experience: > 3 months.	Double-blind crossover trial	CHO	16 g	Immediately prior and during exercise.	– Performance was ↔ in FGB on both trials.
Howarth et al. [87]	11 CrossFit® athletes; training experience: NR.	Crossover trial	CHO	Ad libitium	During the workout.	– Performance and RPE were ↔ in both groups.
Kramer et al. [88]	12 male CrossFit® athletes; training experience: > 4 months.	Double-blind crossover trial	Nitrate	8 mmol·d^−1^	6 days. Two times per day: morning and evening.	– No differences between groups were noted for CrossFit® performance (Grace and 2 km rowing). – The trend was maintained for physical performance with the exception of Wingate protocol. – Substantial improvements were noted for the $NO_{3}^{-}$ group (6.6%) on peak power output.
Outlaw et al. [89]	29 male and females CrossFit® participants; training experience: > 6 months.	Crossover	Pre-workout: tart cherry extract, beet root Extract, green tea and black tea extract. Post-workout.	Pre-workout: 19 g Post-workout: females (20 g protein, 40 g CHO), males (40 g protein, 80 g CHO).	6 weeks: prior to the session.	– Performance on Wingate mean power and VO_2max_ ↑ in the supplementation group. – CrossFit® performance↑ on supplementation group but, without statistical significance.
Jacobs et al. [90]	16 male and females CrossFit® participants; training experience: > 6 months.	Crossover	Commercial pre-workout supplement with beta-alanine, grape seed extract, coenzyme Q10, brown rice extract, natural caffeine, vitamins and minerals.	NR	20 minutes prior to Cindy workout.	– Cindy performance ↑ in pre-workout supplement.

### 3.3. Results of individual studies and meta-analysis

#### 3.3.1. Energy and nutritional intake

**Figure 2.**
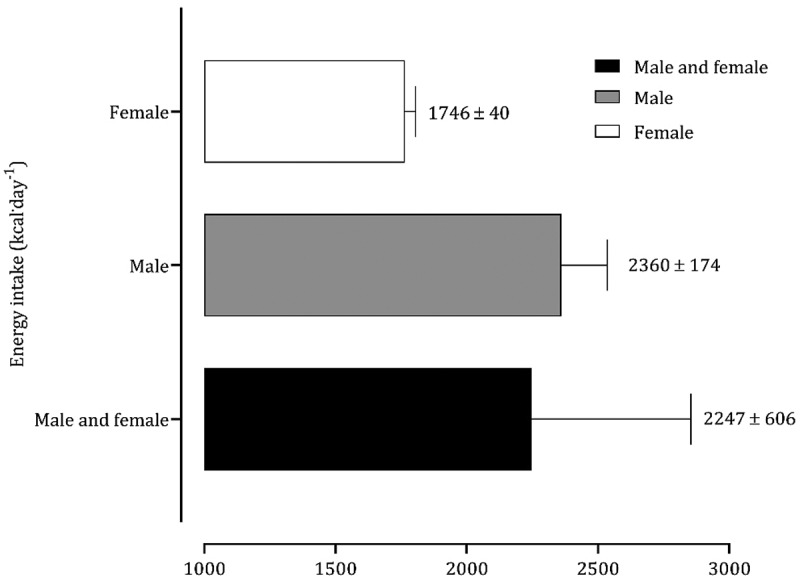
Mean energy intake of studies with CrossFit® participants.

**Figure 3.**
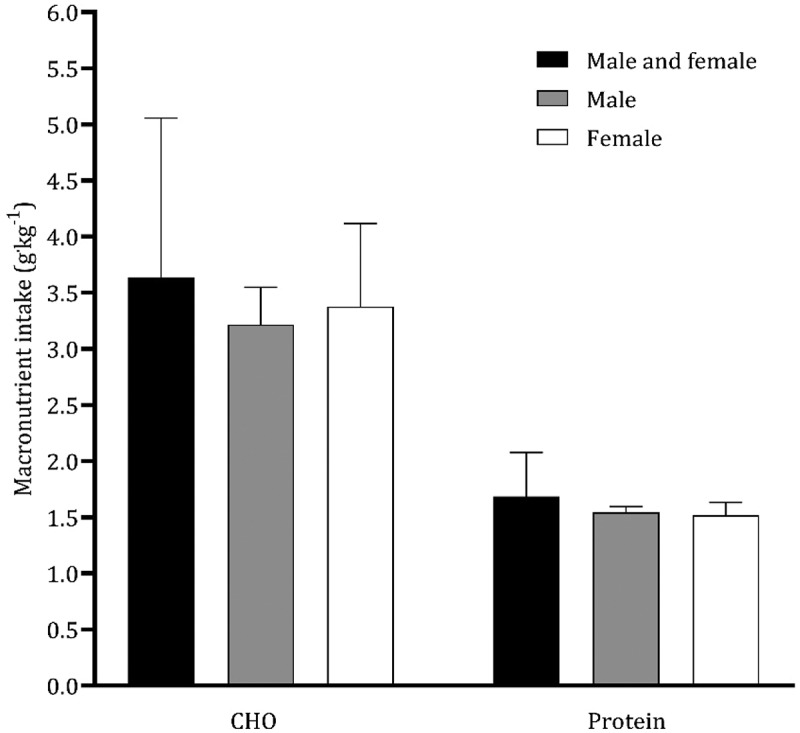
Mean CHO and protein intake of studies with CrossFit® participants.

**Figure 4.**
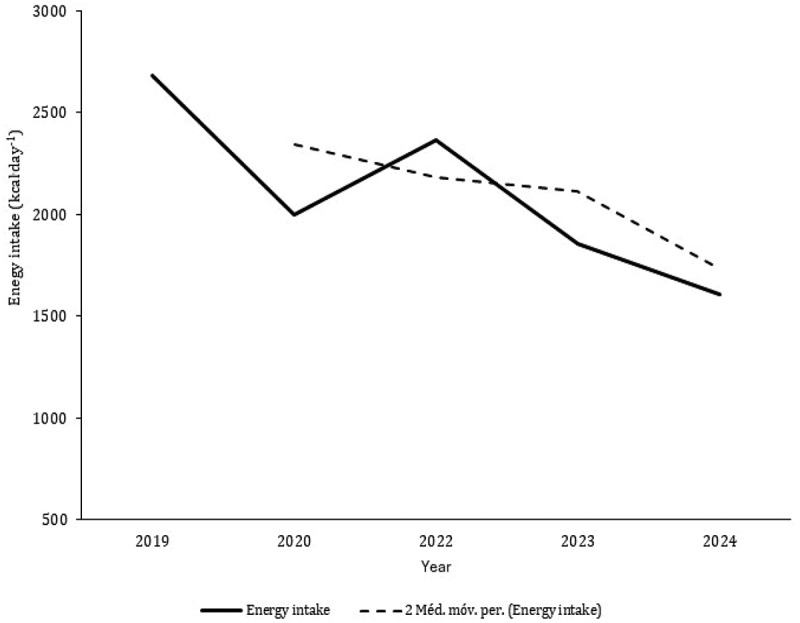
Mean values of energy intake and moving averages plot against the year of publication of the included studies (De Jesus et al. [34]; Rezende et al. [37]; Pearson and Jenkins [41]; gonçalves et al. [42]; Vieira et al. [45]; Gogojewicz et al [48]; Zaykova [49]).

**Figure 5.**
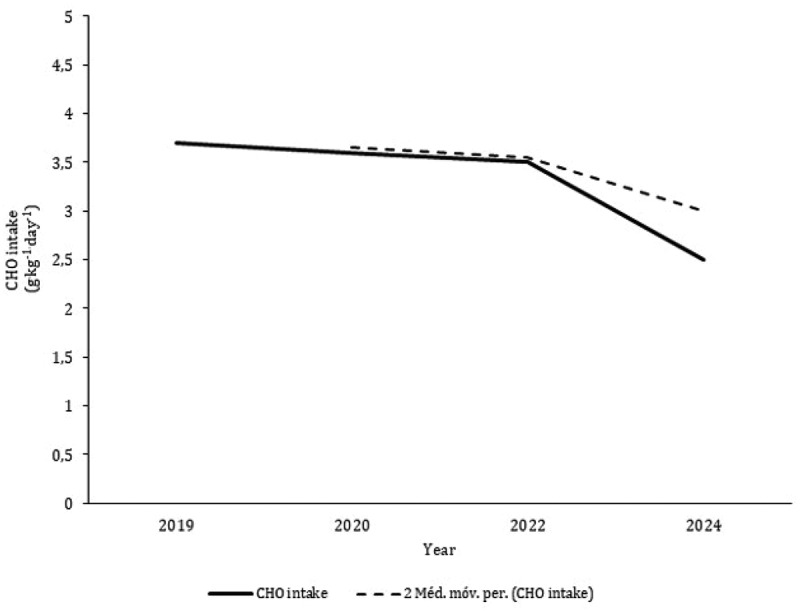
Mean values of CHO intake and moving averages plot against the year of publication of the included studies (De Jesus et al. [34]; Pearson and Jenkins [41]; gonçalves et al. [42]; Vieira et al. [45]; Gogojewicz et al. [48]; Zaykova [49]; Fayad [52]).

**Figure 6.**
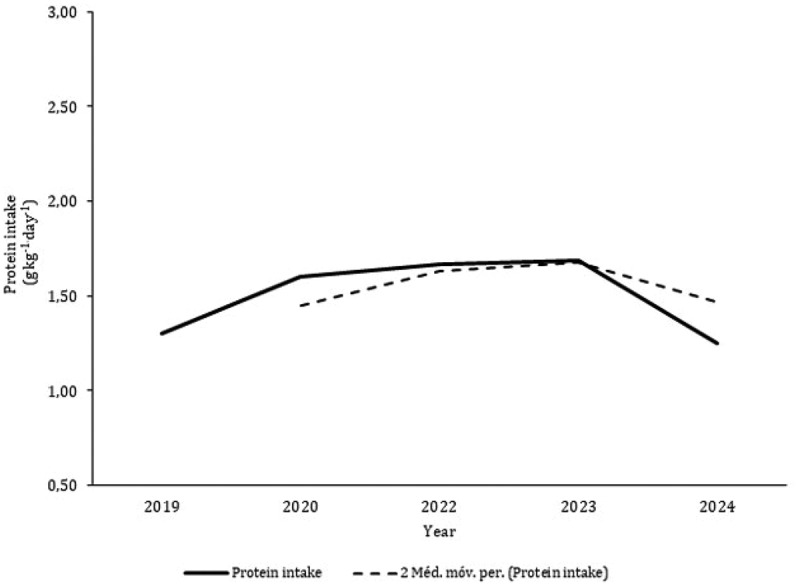
Mean values of protein intake and moving averages plot against the year of publication of the included studies (De Jesus et al. [34]; Rezende et al. [37]; Pearson and Jenkins [41]; gonçalves et al. [42]; Vieira et al. [45]; Gogojewicz et al [48]; Zaykova [49]; Fayad [52]).

Nine studies [34,37,41,42,45,48,49,52,55] aimed to analyze nutritional and dietary intake. Data from 10 CrossFit^Ⓡ^ participants with six months of experience were reported as medians and, consequently, are not included in the figures [55]. The average and standard deviation of energy intake data points extracted from the literature combining male and female participants revealed a mean ingestion of 2247 ± 606 kcal.day^−1^ (Figure 2). Although only two studies exclusively included female participants [41,48], the intake of females (1746 ± 40 kcal.day^−1^) is substantially lower than that of male CrossFit^Ⓡ^ participants (2360 ± 174 kcal.day^−1^). CHO intake was greater in females than in males (females: 3.4 g.kg^−1.^day^−1^; males: 3.2 g.kg^−1.^day^−1^), but protein ingestion was comparable in both sexes (Figure 3). The average intakes of CHO and protein in both groups were 3.6 g.kg^−1.^day^−1^ and 1.7 g.kg^−1.^day^−1^, respectively. The moving averages of energy and CHO tended to decrease from 2019 to 2024 (Figures 4 and 5). Protein intake has remained reasonably stable over the last five years (Figure 6).

**Figure 7.**
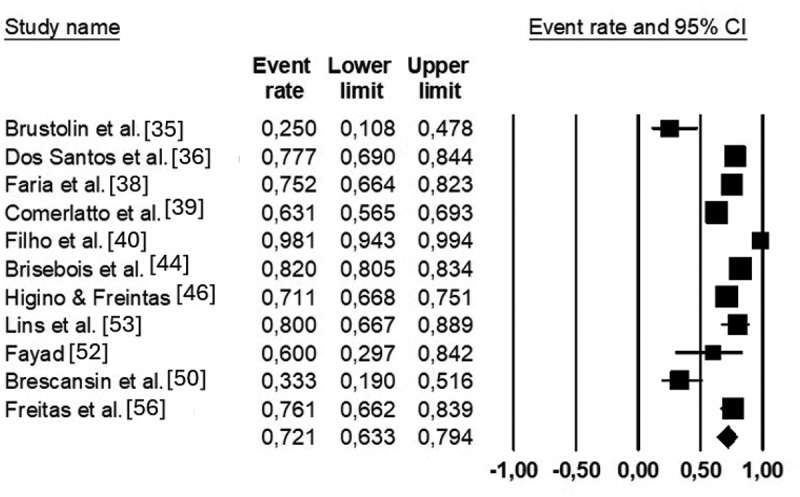
Meta-analysis of the use of ergogenic aids among CrossFit® participants.

#### 3.3.2. Percentage of use of ergogenic aids

The prevalence of supplement use ranged from 25% to 98%. The mean prevalence was 72% (95% CI: 63% to 79%). The I^2^ was 93%, which indicates that 93% of the observed variance in effects is real (Figure 7). Creatine (range: 26% to 61%) and protein (range: 100% to 28%) were the most prevalent ergogenic aids ingested by CrossFit^Ⓡ^ participants in seven studies [21,38–40,44,46,50,52,53,56].

#### 3.3.3. Dietary interventions

**Figure 8.**
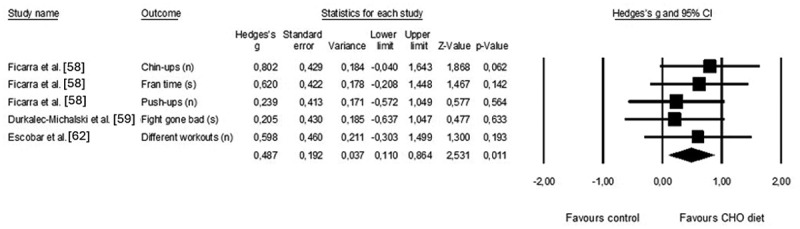
Forest plot of effects of CrossFit® performance after the implementation of CHO or customary diets. The black diamond reflects the overall result.

Three different diets were considered in the analysis: high CHO [58], vegan [59], and Mediterranean [62]. Positive Hedges’s g values indicate the benefits of CHO diets. Three studies provided data on the effects of diets with a greater percentage of CHO than that of customary diets on CrossFit^Ⓡ^ performance (pooled *n* = 31; Figure 8). The effect of CHO diets on CrossFit^Ⓡ^ performance was significantly moderate (Hedge’s g = 0.487; 95% CI: 0.110 to 0.886). No trimmed studies were identified, and Egger’s regression intercept was nonsignificant (Egger’s intercept = 5.44, *p* = 0.563), indicating no risk of publication bias. Heterogeneity was low. Two studies with different designs (i.e. crossover and parallel) investigated the effects of a ketogenic diet on aerobic outputs assessed in the laboratory [61] and CrossFit^Ⓡ^ performance [60].

Data for the ketogenic diet were limited to two studies [60,61]. The former tested the effects of diet on laboratory outcomes ($\dot{V}{O_{2}}_{\max}$), time to exhaustion, and maximal power), whereas the latter focused on CrossFit^Ⓡ^ performance (maximal repetition squat, maximal repetition power clean, number of push-ups, 400-m running). The mean differences between the ketogenic and customary diets did not indicate a positive effect of the ketogenic diet on performance. Given the differences in the outcomes between studies, conducting a meta-analysis for the ketogenic diet was impossible.

#### 3.3.4. Ergogenic aid interventions

Considering the number of studies and outcomes analyzed, the meta-analysis was feasible for CHO and caffeine.

**Figure 9.**
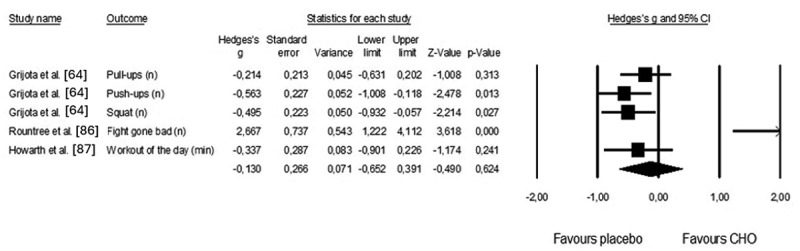
Forest plot of effects of CrossFit® performance considering CHO or placebo intake prior or during the workout. The black diamond reflects the overall result.

One study of caffeine [65] reported multiple comparisons (low-dose vs. medium-dose vs. high-dose vs. placebo) for three outcomes of CrossFit^Ⓡ^ performance (repetitions on Fight Gone Bad round 1, repetitions on Fight Gone Bad round 2, repetitions on Fight Gone Bad round 1, repetitions on Fight Gone Bad round 3). The average value of the medium dose was considered for the analyses, and the effect sizes of three rounds collapsed. Four crossover studies examined the impact of caffeine ingestion prior to the workout on CrossFit^Ⓡ^ performance (pooled *n* = 73). Overall, the effects of caffeine on performance are moderate (Hedges’ g = 0.371) but not significant. Two trimmed studies were identified, and the adjusted Hedge’s value decreased to 0.144 (95% CI: −0.409 to 0.696), which is interpreted as a small effect. Egger’s regression intercept did not identify the risk of biased publication (Egger’s intercept = 2.506, *p* = 0.05) (Figure 9).

**Figure 10.**
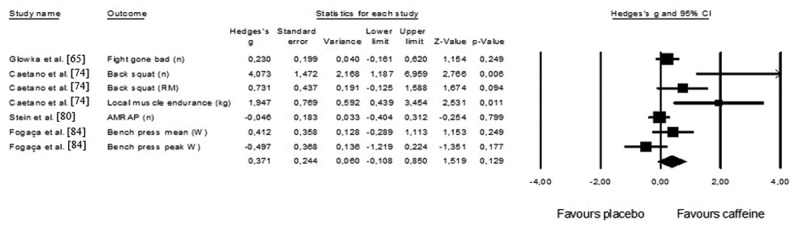
Forest plot of effects of CrossFit® performance considering caffeine or placebo intake prior to the workout. The black diamond reflects the overall result.

Three crossover studies investigated the effects of CHO intake prior to and/or during the workout on CrossFit^Ⓡ^ performance (pooled *n* = 40). CHO ingested did not affect physical performance (Hedges’ g = −0.130; 95% CI: −0.652 to 0.391, *p =* 0.624), as shown in Figure 10. One trimmed study was identified when the Hedge’s g values were plotted against the standard error. The adjusted Hedges’ value for random effects was 0.063 (95% CI: − 0.492 to 0.613). The risk of publication bias was identified for CHO intake (Egger’s intercept = 5.489, *p* = 0.003).

AMRAP (as many rounds as possible).

Sodium bicarbonate supplementation prior to CrossFit^Ⓡ^ performance was examined in three studies [67,72,79], but the findings across these studies were inconsistent. One study reported no significant differences in Fran time or the time to complete the 500-m rowing test between the sodium bicarbonate and placebo groups [67]. However, sodium bicarbonate supplementation positively affected both time and power during the 2 km rowing test [72] as well as several repetitions performed during Cindy’s workout [79].

Scientific evidence about these and other ergogenic aids used near training or competition (e.g. nitrate, beetroot juice) to optimize performance is limited. Only three studies focused on the impact of ergogenic aids on recovery [66,76,84].

#### 3.3.5. Risk of bias

The risk of bias in each study indicated that the power sampling calculation was not reported in 19 studies. Twenty-two studies were cross-sectional, the exposures were not measured over time, and most assessors were aware of the participants’ exposure (18 studies). In five studies, the blinding of assessors could not be determined. The PEDro scale of the interventional studies ranged from 5 to 11 points. Sixteen studies did not present or found significant differences between groups at baseline, which can influence the outcomes, and 11 records did not clearly explain the eligibility criteria used to recruit CrossFit^Ⓡ^ participants (Supplementary Material 2). Overall, 19 studies were classified as excellent, 9 studies as good and 4 studies as fair.

## 4. Discussion

This review summarizes the evidence concerning nutrition and supplementation use among CrossFit^Ⓡ^ participants and identifies potential gaps to guide future research. Relevant findings emerged from the current scoping review: (1) energy intake in CrossFit^Ⓡ^ participants is substantially lower in females than in males, although studies focused exclusively on females are limited; (2) the intake of CHO is below the recommendations for athletes, whereas the ingestion of protein is adequate; (3) CrossFit^Ⓡ^ participants tend to neglect CHO intake; (4) the use of ergogenic aids is considerable, with creatine and protein being the most reported supplements to optimize performance and recovery, respectively; (5) analyses of diets with higher contents of CHO or fat (i.e. a ketogenic diet) show that CHO has a moderate effect on CrossFit^Ⓡ^ performance, whereas a ketogenic diet does not affect performance; (6) considerable research using different supplements has been conducted in CrossFit^Ⓡ^ participants; however, only the data on caffeine and CHO are to generate an effect size on performance; and (7) the evidence concerning the effects of supplements on recovery is limited and warrants future research.

### 4.1. Energy and nutritional intake

Achieving adequate daily energy and nutrient requirements in sports is crucial for optimizing exercise performance, manipulating body composition, and facilitating recovery [18]. However, the definition of guidelines for CrossFit^Ⓡ^ practitioners is particularly challenging because only one study reported the total daily energy expenditure based on data derived from a heart rate monitoring device [48]. The latter study estimated daily energy expenditures of 2598 kcal.day^−1^ and 2828 kcal.day^−1^ for female and male participants, respectively [48]. Compared with the overall mean daily energy intake found in the current review, we report negative energy balances of −468 kcal.day^−1^ and −852 kcal.day^−1^ for males and females, respectively. A negative energy balance was noted in endurance runners [91], but these values could also be explained by the limitations of the instruments used to estimate energy intake [92,93]. A meta-analysis of 11 studies used doubly labeled water to examine the accuracy of self-reported energy intake in athletes and reported that daily energy intake was underreported by 19% [93]. The studies reporting energy intake included in the present review used 24-hour recall [34,37,52], food diaries [42,45,48] or questionnaires [41]. Taking into account the daily energy expenditure reported in Polish CrossFit^Ⓡ^ participants as a reference [48], adjusting the mean total energy intake of the current review by 19% results in an energy balance of −20 kcal for males, whereas a significant negative energy balance persists for females (−520 kcal). These findings suggest that individuals who follow female CrossFit^Ⓡ^ participants or athletes must frequently assess their energy intake, energy expenditure, body weight, and body composition to avoid periods of chronic energy restriction.

CrossFit^Ⓡ^ participants did not meet the nutritional recommendations of CHO specified for athletes [13,18]. Considering the range of 6–12 g.kg^−1.^day^−1^ CHO, the mean values of the current review are below the lower limit of the guidelines [94]. Although the lower intake of CHO differs between sports [91,95], this issue is particularly alarming in CrossFit^Ⓡ^ because high-intensity workouts characterize sessions. Nevertheless, the lower values of CHO identified in this review were expected because the recommendations for CrossFit^Ⓡ^ participants promoted high protein intake and low CHO ingestion [96]. The decrease in CHO intake has become more pronounced over time. Thus, the impacts of nutritional guidelines for CrossFit^Ⓡ^ participants on muscle and hepatic glycogen need to be reviewed to optimize performance and recovery [20,97]. Given the variability in exercise load, volume and rest among CrossFit^Ⓡ^ workouts, the quantities of CHO that should be ingested in different workouts need to be determined. Using the same participants, the maximal oxygen uptakes for the Cindy (complete as many rounds as possible in 20 minutes: 5 pull-ups, 10 push-ups, 15 air squats) and Fran (complete 21-15-9 repetitions for time: thrusters, pull-ups) workouts were 34 and 29 ml.kg^−1^.min^−1^, respectively [7], whereas the mean heart rate on a workout that focused on complete rounds for time was substantially higher than that of a challenge where participants should perform as many rounds as possible in 5 minutes [98]. The metabolic demands of different workouts vary significantly, and appropriate quantities of energy and CHO should be ingested based on the type of session and the phase of the season following the concept of nutritional periodization available for endurance and power sports [99–102]. The current review does not provide specific guidelines for CHO intake among CrossFit^Ⓡ^ participants. Given the high intensity of CrossFit^Ⓡ^ workouts, athletes should consider increasing their CHO consumption to optimize performance and recovery. Additionally, it is important for athletes to be familiar with adequate sources of CHO.

Regarding protein ingestion, the *International Society of Sports Nutrition* recommends intakes of 1.4–2.0 g.kg^−1^ for maintaining and building muscle mass [103], whereas 1.6 g.kg^−1^ is defined as the upper cutoff value to promote changes in fat-free mass [104]. The mean protein intake reported in this review is consistent with the recommended values. CrossFit^Ⓡ^ participants and nutritionists should be aware that greater fat mass can affect performance [105,106] and discriminate athletes by competitive level [105]. Additional questions, such as the type, timing, and distribution of protein intake across the day, warrant further research. Given the importance of protein intake, it is unsurprising that it has been reported as one of the most prevalent ergogenic aids ingested by CrossFit^Ⓡ^ participants.

### 4.2. Prevalence of ergogenic aids

The meta-analysis of the prevalence of ergogenic aid usage revealed an average intake of ~ 72%. The most reported supplements are protein and creatine, with the aim of optimizing recovery and performance. The benefits of creatine supplementation are associated with increased muscle phosphocreatine and regeneration of phosphocreatine during exercise recovery [107]. The short- and long-term benefits of creatine supplementation on strength and resistance activities are well documented in the literature, as are the timing and dosage necessary for athletes [108,109]. Therefore, it is unsurprising that creatine has been reported as one of the most preferred supplements used by CrossFit^Ⓡ^ participants. One potential disadvantage of creatine is in body weight due to intracellular water retention [107]; however, gains in body weight could be particularly beneficial in supporting the extreme loads imposed by CrossFit^Ⓡ^.

The type of protein ingested was reported in three studies [38–40], with whey protein being the most commonly used protein by CrossFit^Ⓡ^ participants. Whey protein is rapidly digestible and contains a high proportion of essential amino acids (including leucine), which determines the protein quality to optimize muscle protein synthesis and body composition [103,110]. The attainment of protein intake guidelines for resistance training and skeletal muscle hypertrophy adaptations, independent of the timing and quality, has not been compared between whey protein and other types of protein [111]. Therefore, supplementation with whey protein in conjunction with dietary sources of protein is central among CrossFit^Ⓡ^ practitioners [110,112].

### 4.3. Dietary interventions

The recommended dietary prescription for CrossFit^Ⓡ^ participants is to follow two different types of diets: the Zone and the Paleo diets. The Zone Diet is characterized by meals and snacks with a macronutrient distribution of 40% CHO, 30% protein, and 30% fat, whereas the Paleo Diet recommends the ingestion of fruits, vegetables, lean meats, and fish and the avoidance of dairy foods, legumes, and grains with a macronutrient distribution of 35–45% CHO, 20–35% protein, and the remaining percentage from fat. Both dietary approaches are characterized by a lower ingestion of CHO and an increased percentage of protein [113]. These diets lack a scientific basis for CrossFit^Ⓡ^. As such, dietary prescriptions among CrossFit^Ⓡ^ participants need further evidence to support their use.

The Atkins diet is classified as a nonketogenic, low-carbohydrate diet, and the potential role of this dietary approach is to deplete glycogen stores, increase fat oxidation, and optimize gluconeogenesis [113]. The ketogenic diet, characterized by relatively high levels of fat intake, may impact on CrossFit^Ⓡ^ performance [60,61] via a mechanism similar to that of the Atkins diet: it minimizes glycogen stores, maximizes fat oxidation, and provides ketone bodies as a potential substrate for muscle tissue and the central nervous system [114]. Recently, the increase in fat oxidation stimulated by a ketogenic diet was demonstrated to be due to an increase in the oxygen cost (i.e. less efficiency) without increasing performance among elite racewalkers [115]. Because CHO metabolism results in a greater ratio of reducing NADH to FADH_2_ than fat does, more energy is produced per unit of oxygen when CHO are used as fuel in the electron transport chain [116]. The presence of oxygen and other factors (e.g. availability of coenzyme A, carnitine palmitoyl transferase) are a determinant and a critical factor in explaining the shift of CHO and fats in endurance events [117]. Moreover, at high intensities, oxygen cannot be used; consequently, the metabolism of fat via beta-oxidation is not considered. Therefore, ketogenic diets for athletes in high-intensity sports, such as CrossFit^Ⓡ^, did not significantly improve performance outputs compared with those of the control groups [60,61]. Another issue associated with the ketogenic diet is the difficulty of participants adhering to the diet [118].

In contrast to a ketogenic diet, applying dietary prescriptions with a higher percentage of carbohydrates was moderately beneficial for CrossFit^Ⓡ^ performance outputs. Although the focus of a vegan diet is the amount of protein ingested (1.5–2.0 g.kg^−1.^day^−1^), the study that examined the effects of this type of diet on performance and biomarkers was classified as a diet with a relatively high content of CHO. CHO were reported to be ingested at 4.5–5.5 g.kg^−1.^day for a vegan diet [60]. Diets focused predominantly on CHO appear to satisfy athletes’ energy needs, support high-intensity activities, and improve body composition [113].

### 4.4. Acute strategies

Many supplements have been used before or during workouts by CrossFit^Ⓡ^ participants. Most supplements lack scientific evidence, and only two studies present sufficient data on the potential effects on performance: CHO and caffeine.

The ergogenic effects of carbohydrates have been considered during exercise because they represent a nutritional strategy that delays glycogen depletion and improves performance [119,120], and the recommendations for CHO ingestion during exercise consider the time spent exercising. For example, in a soccer match, the ingestion of 30–60 g.hour^−1^ or 60 g of carbohydrates before each half is recommended [121,122], whereas in endurance sports, 60 g.hour^−1^ is advised in efforts >2.5 hours [20]. This review did not confirm the effect of CHO on CrossFitⓇ performance. Two studies [64,86] examined the effects of carbohydrate ingestion in short-performance protocols. These results suggest that athletes may use muscle and hepatic glycogen from carbohydrates ingested during the hours before exercise to complete workouts. In other words, the protocols used did not allow the depletion of glycogen stores. A third study considered a long workout [86]; however, the intake of carbohydrates occurred during the workout. Consequently, future studies should investigate the ingestion of carbohydrates during CrossFit^Ⓡ^ challenges, taking into account the duration of exercise and intensity. Additional questions about carbohydrate supplementation include tolerance to higher quantities [20,123] and practical application during high-intensity workouts. Consequently, further research should consider testing the impact of carbohydrates within training sessions and not solely on a specific part of the workout or between competitions that occur on the same day. In fact, the effects of carbohydrates on resistance training tend to be positive, particularly when training sessions exceed 45 minutes [124]. Carbohydrate intake can be especially beneficial for athletes who engage in longer training periods or have multiple training sessions in a single day.

Caffeine is a popular ergogenic aid in the context of sports [125]. The effect of caffeine ingestion on CrossFit^Ⓡ^ performance was small and nonsignificant. Nevertheless, the results were consistent with a previous meta-analysis that examined the effects of the ingestion of moderate doses (3–6 mg.kg^−1^) on the mean power output and time to complete a trial. However, both analyses in this study noted significant effects [126]. A combination of 20 studies also revealed that caffeine ingestion significantly improved strength (1 repetition maximum test) and power (vertical jump) outputs [127]. A narrative review also highlighted the positive effect of caffeine intake on muscular endurance, strength, and power in a resistance context [128]. The nonsignificant effects found in this review could be explained by the demands of CrossFit^Ⓡ^, which requires endurance, strength, and power capacities.

The study design, dosage, timing, and formula can also affect the caffeine response [129]. The placebo effect was noted in a study of 48 cyclists, with those who received a placebo and thought they ingested caffeine, improving exercise performance [130]. Consequently, ensuring the blinding of participants, which was guaranteed in the four studies included in this meta-analysis, is important. Although the timing and dosage are two factors that need further investigation, the quantity and timing of caffeine ingested by samples included in the meta-analysis ranged from 3 to 6 mg.kg^−1^ and from 60 to 70 minutes, respectively. The dosage followed the recommendations for caffeine intake, with lower dosages (2 mg.kg^−1^) having no effect on exercise performance [131]. Higher dosages of caffeine should be considered in maximal repetition efforts [132]. This issue warrants further study in CrossFit^Ⓡ^ participants because muscular strength and endurance are central to improving performance. The ingestion of caffeine 60 minutes before exercise is widely accepted to achieve the ergogenic effects of this supplement [133]. Nevertheless, the caffeine response depends on genetic and epigenetic factors (e.g. sex, habitual caffeine use, smoking) [134].

### 4.5. Limitations and future research

The present study provides a broad overview of the nutritional evidence in CrossFit^Ⓡ^, but some limitations should be recognized: (1) Exercise and daily energy expenditure reference values are scarce among CrossFit^Ⓡ^ participants. Nevertheless, the assessment of exercise energy expenditure is challenging because the error associated with wearable monitors ranges from 15.1% to 57.0% [135]. (2) The meta-analysis testing the effects of carbohydrates and caffeine used a limited number of studies. (3) Multiple ergogenic aids were used among CrossFit^Ⓡ^ participants, but robust conclusions could not be drawn given the variability in outcomes and interventions. For example, a well-designed, double-blind, randomized controlled trial revealed beneficial effects of sodium bicarbonate on CrossFit^Ⓡ^ performance (time trial on a 2 km rowing test); however, the data are limited to one study, and the authors determined the peak blood sodium bicarbonate for each participant [72]. Among the 17 participants, sodium bicarbonate improved the Fran time and time to complete 500 m rowing [67], whereas no effects were found in performance for Cindy’s workout [79]. These inconsistent findings indicate that the differences in the effects of sodium bicarbonate on performance and recovery between training sessions or competitions need further investigation. (4) Few studies have focused on recovery, and this issue needs particular attention given the high volume and intensity of training exposure in CrossFit^Ⓡ^. The studies included in this review have limitations that should not be ignored: sample size and the definition of a CrossFit^Ⓡ^ participant or athlete. The training characteristics of individuals from different competitive levels are not equivalent (see Supplementary Material 3 to compare training sessions of beginner, intermediate, and advanced practitioners of CrossFit^Ⓡ^). This issue must be considered when the eligibility criteria are defined [136].

## 5. Conclusions and practical applications

The present scoping review provides new insights into nutrition and CrossFit^Ⓡ^, which might yield changes in practice. Although the ingestion of protein was appropriate, CHO consumption was below the recommended limit for athletes, and CHO consumption has decreased in recent years. Nutritionists need to educate coaches and athletes about the importance of CHO in high-intensity sporting activities, such as CrossFit^Ⓡ^. Diets with high CHO values show performance benefits, and recommendations surrounding the Zone or Paleo diet need to be revised. CHO intake is essential for optimizing performance, whereas protein intake can influence body composition, which, in turn, may affect performance. Therefore, athletes should pay special attention to the ingestion of these two macronutrients by managing their intake effectively. Data available on the ketogenic diet remain limited, although the existing studies do not demonstrate performance benefits. The use of nutritional aids is common among CrossFit^Ⓡ^ participants, with creatine and protein being the most reported. The benefits of these nutritional aids for performance and recovery are well documented, and consequently, they should be incorporated into the regimen of CrossFit^Ⓡ^ participants. Given the variability of CrossFit^Ⓡ^ workouts, future studies examining the long-term impact of creatine on performance and protein on recovery are needed. When consumed acutely, CHO had no effect on performance; however, ingesting 6 mg.kg^−1^ caffeine 60–70 minutes before exercise had a small effect on performance. In summary, monitoring carbohydrate and protein intake is essential to determine whether CrossFit^Ⓡ^ participants meet the recommended guidelines. Regarding the scientific evidence surrounding supplements, both creatine and protein should be consumed, while caffeine may be utilized prior to training sessions.

## Supplementary Material

Supplemental Material

Supplemental Material

Supplemental Material

## Data Availability

The data that support the findings of this study are available from the corresponding author, DVM, upon reasonable request.
