# Supplementary material for: Nutrition in CrossFit® – scientific evidence and practical perspectives: a systematic scoping review
Source: J Int Soc Sports Nutr. 2025 Jun 5;22(1):2509674. doi: 10.1080/15502783.2025.2509674 (PMC12143013; doi:10.1080/15502783.2025.2509674)
Supplement: Supplemental Material [file RSSN_A_2509674_SM9484.docx]

**Supplementary Material 1.** Full search strategy for each database.

| PubMed |  | (("nutrition*or"[All Fields] AND "nutritional strategy"[All Fields]) OR "nutritional intervention"[All Fields] OR ("diet*"[All Fields] AND ("carbohydrate s"[All Fields] OR "carbohydrated"[All Fields] OR "carbohydrates"[MeSH Terms] OR "carbohydrates"[All Fields] OR "carbohydrate"[All Fields])) OR ("glucose"[MeSH Terms] OR "glucose"[All Fields] OR "glucoses"[All Fields] OR "glucose s"[All Fields]) OR ("protein s"[All Fields] OR "proteinous"[All Fields] OR "proteins"[MeSH Terms] OR "proteins"[All Fields] OR "protein"[All Fields]) OR ("collagen"[MeSH Terms] OR "collagen"[All Fields] OR "collagens"[All Fields] OR "collagen s"[All Fields] OR "collagenation"[All Fields] OR "collagene"[All Fields] OR "collageneous"[All Fields] OR "collagenic"[All Fields] OR "collagenization"[All Fields] OR "collagenized"[All Fields] OR "collagenous"[All Fields]) OR "fat"[All Fields] OR "ketone*"[All Fields] OR "antioxidant*"[All Fields] OR ("vitamin s"[All Fields] OR "vitamine"[All Fields] OR "vitamines"[All Fields] OR "vitamins"[Pharmacological Action] OR "vitamins"[MeSH Terms] OR "vitamins"[All Fields] OR "vitamin"[All Fields]) OR "polyphenol*"[All Fields] OR ("fruit"[MeSH Terms] OR "fruit"[All Fields] OR "fruits"[All Fields] OR "fruit s"[All Fields] OR "fruited"[All Fields] OR "fruiting"[All Fields]) OR ("creatine"[MeSH Terms] OR "creatine"[All Fields] OR "creatin"[All Fields] OR "creatines"[All Fields]) OR ("caffein"[All Fields] OR "caffeinated"[All Fields] OR "caffeine"[MeSH Terms] OR "caffeine"[All Fields] OR "caffeine s"[All Fields] OR "caffeines"[All Fields] OR "caffeinism"[All Fields]) OR "nitrate*"[All Fields] OR ("beetroot"[All Fields] OR "beetroots"[All Fields]) OR "tart cherry"[All Fields] OR ("beta alanine"[MeSH Terms] OR "beta alanine"[All Fields] OR ("beta"[All Fields] AND "alanine"[All Fields]) OR "beta alanine"[All Fields]) OR ("sodium bicarbonate"[MeSH Terms] OR ("sodium"[All Fields] AND "bicarbonate"[All Fields]) OR "sodium bicarbonate"[All Fields]) OR ("supplement*or"[All Fields] AND "energy*"[All Fields]) OR "macronutrient*"[All Fields] OR "micronutrient*"[All Fields] OR "mineral*"[All Fields] OR "electrolyte*"[All Fields]) AND "CrossFit"[All Fields] |
| --- | --- | --- |
| Scopus |  | ALL ( ( ( nutrition* OR "nutritional strategy" OR "nutritional intervention" OR diet*, AND carbohydrate OR glucose OR protein OR collagen OR fat OR ketone* OR antioxidant* OR vitamin OR polyphenol* OR fruit OR creatine OR caffeine OR nitrate* OR beetroot OR "tart cherry" OR beta AND alanine OR sodium AND bicarbonate OR supplement* OR energy* OR macronutrient* OR micronutrient* OR mineral* OR electrolyte* ) AND crossfit ) ) |
| SPORTDiscus |  | ((nutrition* OR “nutritional strategy” OR “nutritional intervention” OR diet*, carbohydrate OR glucose OR protein OR collagen OR fat OR ketone* OR antioxidant* OR vitamin OR polyphenol* OR fruit OR creatine OR caffeine OR nitrate* OR beetroot OR “tart cherry” OR beta alanine OR sodium bicarbonate OR supplement* OR energy* OR macronutrient* OR micronutrient* OR mineral* OR electrolyte*) AND CrossFit) |
| Web of Science |  | ((nutrition* OR “nutritional strategy” OR “nutritional intervention” OR diet*, carbohydrate OR glucose OR protein OR collagen OR fat OR ketone* OR antioxidant* OR vitamin OR polyphenol* OR fruit OR creatine OR caffeine OR nitrate* OR beetroot OR “tart cherry” OR beta alanine OR sodium bicarbonate OR supplement* OR energy* OR macronutrient* OR micronutrient* OR mineral* OR electrolyte*) AND CrossFit) (All Fields) |
