# Supplementary material for: Nutrition in CrossFit® – scientific evidence and practical perspectives: a systematic scoping review
Source: J Int Soc Sports Nutr. 2025 Jun 5;22(1):2509674. doi: 10.1080/15502783.2025.2509674 (PMC12143013; doi:10.1080/15502783.2025.2509674)
Supplement: Supplemental Material [file RSSN_A_2509674_SM9483.docx]

**Supplementary Material 2.** Tools and respective assessment of risk of bias considering the two types of study designs.

| Criteria no. | Question |
| --- | --- |
| 1 | Was the research question or objective in this paper clearly stated? |
| 2 | Was the study population clearly specified and defined? |
| 3 | Was the participation rate of eligible persons at least 50%? |
| 4 | Were all the subjects selected or recruited from the same or similar populations (including the same time period)? Were inclusion and exclusion criteria for being in the study prespecified and applied uniformly to all participants? |
| 5 | Was a sample size justification, power description, or variance and effect estimates provided? |
| 6 | For the analyses in this paper, were the exposure(s) of interest measured prior to the outcome(s) being measured? |
| 7 | Was the timeframe sufficient so that one could reasonably expect to see an association between exposure and outcome if it existed? |
| 8 | For exposures that can vary in amount or level, did the study examine different levels of the exposure as related to the outcome (e.g., categories of exposure, or exposure measured as continuous variable)? |
| 9 | Were the exposure measures (independent variables) clearly defined, valid, reliable, and implemented consistently across all study participants? |
| 10 | Was the exposure(s) assessed more than once over time? |
| 11 | Were the outcome measures (dependent variables) clearly defined, valid, reliable, and implemented consistently across all study participants? |
| 12 | Were the outcome assessors blinded to the exposure status of participants? |
| 13 | Was loss to follow-up after baseline 20% or less? |
| 14 | Were key potential confounding variables measured and adjusted statistically for their impact on the relationship between exposure(s) and outcome(s)? |
|  | **Quality Rating (Good, Fair, or Poor)** |

**Legend:**

1-YES 2 - NO; 3 - cannot determine, not applicable or not reported

| Study | Quality Assessment Tool for Observational Cohort and Cross-Sectional Studies checklist question number | | | | | | | | | | | | | |
| --- | --- | --- | --- | --- | --- | --- | --- | --- | --- | --- | --- | --- | --- | --- |
|  | 1 | 2 | 3 | 4 | 5 | 6 | 7 | 8 | 9 | 10 | 11 | 12 | 13 | 14 |
| De Jesus et al. (2024) | 1 | 1 | 3 | 1 | 2 | 1 | 3 | 1 | 1 | 2 | 1 | 2 | 3 | 3 |
| Brustolin et al. (2024) | 1 | 1 | 1 | 1 | 2 | 1 | 1 | 2 | 1 | 2 | 1 | 2 | 3 | 2 |
| Dos Santos et al. (2023) | 1 | 1 | 1 | 1 | 2 | 1 | 1 | 2 | 1 | 2 | 1 | 2 | 3 | 1 |
| Rezende et al. (2023) | 1 | 1 | 1 | 1 | 2 | 1 | 1 | 2 | 1 | 2 | 1 | 2 | 3 | 1 |
| Faria et al. (2023) | 1 | 1 | 1 | 1 | 2 | 1 | 1 | 2 | 1 | 2 | 1 | 2 | 3 | 1 |
| Comerlatto et al. (2023) | 1 | 1 | 1 | 1 | 2 | 1 | 1 | 2 | 1 | 2 | 1 | 2 | 3 | 1 |
| Filho et al. (2023) | 1 | 1 | 1 | 1 | 2 | 1 | 1 | 2 | 1 | 2 | 1 | 2 | 3 | 1 |
| Pearson & Jenkins (2022) | 1 | 1 | 1 | 1 | 2 | 1 | 1 | 2 | 1 | 2 | 1 | 2 | 3 | 1 |
| Gonçalves et al. (2022) | 1 | 2 | 1 | 2 | 2 | 1 | 1 | 2 | 2 | 2 | 2 | 2 | 3 | 1 |
| Mesquita & Cavalcanti (2022) | 1 | 1 | 3 | 1 | 2 | 1 | 1 | 2 | 1 | 2 | 1 | 2 | 3 | 1 |
| Brisebois et al. (2022) | 1 | 1 | 1 | 1 | 2 | 1 | 1 | 1 | 1 | 2 | 1 | 2 | 3 | 1 |
| Vieira et al. (2021) | 1 | 1 | 1 | 1 | 2 | 1 | 1 | 1 | 1 | 2 | 1 | 2 | 3 | 1 |
| Higino & Freitas (2021) | 1 | 1 | 1 | 1 | 1 | 1 | 1 | 1 | 1 | 2 | 1 | 3 | 3 | 1 |
| Kutch (2021) | 1 | 1 | 1 | 1 | 1 | 1 | 1 | 2 | 1 | 1 | 1 | 2 | 3 | 1 |
| Gogojewicz et al. (2020) | 1 | 1 | 1 | 1 | 1 | 1 | 1 | 1 | 1 | 2 | 1 | 2 | 3 | 1 |
| Zaykova (2019) | 1 | 1 | 1 | 1 | 1 | 1 | 1 | 2 | 2 | 2 | 2 | 2 | 3 | 1 |
| Brescansin et al. (2019) | 1 | 1 | 1 | 1 | 2 | 1 | 1 | 2 | 1 | 2 | 1 | 2 | 3 | 1 |
| Terry (2019) | 1 | 1 | 1 | 1 | 2 | 1 | 1 | 2 | 1 | 2 | 1 | 2 | 3 | 1 |
| Fayad (2019) | 1 | 2 | 1 | 1 | 2 | 1 | 1 | 2 | 1 | 2 | 1 | 3 | 3 | 1 |
| Lima et al. (2019) | 1 | 2 | 2 | 1 | 2 | 1 | 1 | 2 | 2 | 2 | 2 | 3 | 3 | 1 |
| Pacheco et al. (2018) | 2 | 2 | 3 | 3 | 2 | 1 | 1 | 1 | 2 | 2 | 2 | 3 | 3 | 1 |
| Bueno et al. (2016) | 1 | 1 | 1 | 1 | 2 | 1 | 1 | 2 | 1 | 2 | 1 | 2 | 3 | 1 |
| Freitas et al. (2016) | 1 | 2 | 1 | 1 | 2 | 1 | 1 | 2 | 2 | 2 | 2 | 3 | 3 | 1 |

Results of methodological quality assessment for Obs1ervational Cohort and Cross-Sectional Studies.

| **PEDro scale** | |
| --- | --- |
| 1. eligibility criteria were specified | |
| 1. subjects were randomly allocated to groups (in a crossover study, subjects were randomly allocated an order in which treatments were received) | |
| 1. allocation was concealed | |
| 1. the groups were similar at baseline regarding the most important prognostic indicators | |
| 1. there was blinding of all subjects? | |
| 1. there was blinding of all therapists who administered the therapy | |
| 1. there was blinding of all assessors who measured at least one key outcome | |
| 1. measures of at least one key outcome were obtained from more than 85% of the subjects initially allocated to groups | |
| 1. all subjects for whom outcome measures were available received the treatment or control condition as allocated or, where this was not the case, data for at least one key outcome was analysed by “intention to treat” | |
| 1. the results of between-group statistical comparisons are reported for at least one key outcome | |
| 1. Were the outcome measures (dependent variables) clearly defined, valid, reliable, and implemented consistently across all study participants? | |
| **Total points** |  |

Questions from the PEDro scale used to evaluate the methodological quality of the interventional studies.

Results of methodological quality assessment for PEDro scale.

| Study | Quality Assessment Tool for PEDro scale | | | | | | | | | | | | Qualitative classification |
| --- | --- | --- | --- | --- | --- | --- | --- | --- | --- | --- | --- | --- | --- |
|  | 1 | 2 | 3 | 4 | 5 | 6 | 7 | 8 | 9 | 10 | 11 | Points |  |
| Grijota et al. (2024) | 1 | 1 | 1 | 1 | 1 | 1 | 1 | 1 | 1 | 1 | 1 | **10** | **Excellent** |
| Główka et al. (2024) | 0 | 1 | 1 | 1 | 1 | 1 | 1 | 1 | 1 | 1 | 1 | **10** | **Excellent** |
| Durkalec-Michalski et al. (2024) | 1 | 1 | 1 | 0 | 1 | 1 | 1 | 1 | 1 | 1 | 1 | **9** | **Excellent** |
| De Souza et al. (2024) | 1 | 1 | 1 | 1 | 1 | 1 | 1 | 1 | 1 | 1 | 1 | **10** | **Excellent** |
| Eroglu et al. (2023) | 1 | 0 | 1 | 0 | 0 | 0 | 0 | 1 | 1 | 1 | 1 | **5** | **Fair** |
| Ziyaiyan et al. (2023) | 1 | 1 | 1 | 1 | 1 | 1 | 1 | 1 | 1 | 1 | 1 | **10** | **Excellent** |
| Mattos et al. (2023) | 0 | 1 | 1 | 1 | 0 | 0 | 0 | 1 | 1 | 1 | 0 | **6** | **Good** |
| Oliveira et al. (2023) | 1 | 1 | 1 | 0 | 1 | 1 | 1 | 1 | 1 | 1 | 1 | **9** | **Excellent** |
| Martin et al. (2023) | 1 | 1 | 1 | 1 | 1 | 1 | 1 | 1 | 1 | 1 | 1 | **10** | **Excellent** |
| Zawieja et al. (2023) | 1 | 1 | 1 | 1 | 1 | 1 | 1 | 1 | 1 | 1 | 1 | **10** | **Excellent** |
| Gomes et al. (2023) | 1 | 1 | 1 | 0 | 0 | 0 | 0 | 1 | 1 | 1 | 1 | **6** | **Good** |
| Caetano et al. (2023) | 1 | 1 | 1 | 0 | 1 | 1 | 1 | 1 | 1 | 1 | 1 | **9** | **Excellent** |
| Silvestre (2023) | 1 | 1 | 1 | 0 | 1 | 1 | 1 | 1 | 1 | 1 | 1 | **9** | **Excellent** |
| Ficarra et al. (2022) | 0 | 0 | 1 | 1 | 0 | 0 | 0 | 1 | 1 | 1 | 1 | **7** | **Good** |
| Fernandez-Lázaro et al. (2022) | 0 | 1 | 1 | 1 | 1 | 0 | 1 | 0 | 1 | 1 | 1 | **8** | **Good** |
| Durkalec-Michalski et al. (2022) | 0 | 1 | 1 | 1 | 0 | 0 | 0 | 1 | 1 | 1 | 1 | **7** | **Good** |
| Durkalec-Michalski et al. (2021) | 1 | 0 | 1 | 0 | 0 | 0 | 0 | 1 | 1 | 1 | 1 | **5** | **Fair** |
| Fernandez-Lázaro et al. (2021) | 0 | 1 | 1 | 1 | 1 | 0 | 1 | 0 | 1 | 1 | 1 | **8** | **Good** |
| Maroufi et al. (2021) | 0 | 1 | 1 | 1 | 1 | 0 | 0 | 1 | 1 | 1 | 1 | **8** | **Good** |
| Toledo et al. (2020) | 1 | 1 | 1 | 0 | 1 | 1 | 1 | 1 | 1 | 1 | 1 | **9** | **Excellent** |
| Stein et al. (2020) | 1 | 1 | 1 | 0 | 1 | 1 | 1 | 1 | 1 | 1 | 1 | **9** | **Excellent** |
| Moro et al. (2020) | 1 | 1 | 1 | 0 | 1 | 1 | 1 | 1 | 1 | 1 | 1 | **9** | **Excellent** |
| Ricordi et al. (2020) | 1 | 0 | 1 | 0 | 1 | 1 | 1 | 1 | 1 | 1 | 1 | **8** | **Good** |
| Garnacho-Castano et al. (2020) | 1 | 1 | 1 | 0 | 1 | 1 | 1 | 1 | 1 | 1 | 1 | **9** | **Excellent** |
| Fogaça et al. (2020) | 1 | 1 | 1 | 1 | 1 | 1 | 1 | 1 | 1 | 1 | 1 | **10** | **Excellent** |
| Durkalec-Michalski et al. (2018) | 1 | 1 | 1 | 0 | 1 | 1 | 1 | 1 | 1 | 1 | 1 | **9** | **Excellent** |
| Kephart et al. (2018) | 0 | 0 | 1 | 0 | 0 | 0 | 0 | 1 | 1 | 1 | 1 | **5** | **Fair** |
| Howarth et al. (2017) | 0 | 1 | 1 | 0 | 0 | 0 | 0 | 1 | 1 | 1 | 0 | **5** | **Fair** |
| Rountree et al. (2017) | 0 | 1 | 1 | 0 | 1 | 1 | 1 | 1 | 1 | 1 | 1 | **9** | **Excellent** |
| Escobar et al. (2016) | 1 | 1 | 1 | 1 | 0 | 1 | 1 | 1 | 1 | 1 | 1 | **9** | **Excellent** |
| Gregory et al. (2016) | 0 | 1 | 1 | 1 | 0 | 0 | 0 | 1 | 1 | 1 | 1 | **7** | **Good** |
| Kramer et al. (2016) | 1 | 1 | 1 | 1 | 1 | 1 | 1 | 1 | 1 | 1 | 1 | **10** | **Excellent** |
|  |  |  |  |  |  |  |  |  |  |  |  |  |  |
