# Supplementary material for: Nutrition in CrossFit® – scientific evidence and practical perspectives: a systematic scoping review
Source: J Int Soc Sports Nutr. 2025 Jun 5;22(1):2509674. doi: 10.1080/15502783.2025.2509674 (PMC12143013; doi:10.1080/15502783.2025.2509674)
Supplement: Supplemental Material [file RSSN_A_2509674_SM9482.docx]

| Level of participant: | **Beginner** |
| --- | --- |
| Warm-up | 3 rounds:  8 cal row  8 reps side deadbug to side plunk clamshell |
| Phase A | Power clean + clean + jerk:  5 sets x 1 +1 +1 80-90% RM, every 2 minutes |
| Phase B | Sumo dedlift:  2 sets × 6 reps 80-90% RM, every 3 minutes |
| Phase C | 4 rounds, each for time:  15 cal assault bike  15 burpee box jump 24 in  20 kettlebell swing 20 kg  2’ minutes rest between each round |
| Phase D | Single arm elbow on knee external rotation:  3 ×10-12 reps, 30-60 sec between sides  Elbow plunk side:  3 × 40-60 sec each side, rest as needed between sets  Single arm kettlebell farmer curry:  accumulate 200 m with 24 kg |

**Supplemental Material 3.** Training sessions of beginner, intermediate and advanced CrossFit® participants.

| Level of participant: | **Intermediate** |
| --- | --- |
| Warm-up | 3 sets (increase the effort each set):  row 1’  bike 1’ |
| Phase A | Lateral jumps over PVC (25’):  5 sets x 10 every 90 sec  minimal contact with floor |
| Phase B | Hang clean:  9 sets × 2 reps 75-85% RM, every 90 sec |
| Phase C | Clean deadlift:  5 sets × 3 reps 89-90% RM, every 3’ |
| Phase D | 4 sets × 30 sec max cal assault bike, every 3’  30 sec: maximal intensity |
| Phase E | Sissy squat  3 × 15-20 reps, rest 60’’  Paralllete L-sit  2’ accumulated |

| Level of participant: | **Advanced** |
| --- | --- |
| **Session 1** | |
| Phase A | Weighted ankle stretch  2 sets × 5 reps  Pullover with hamstring  2 sets × 5 reps |
| Phase B | Seated scapular wall side  2 sets × 5 reps  Belt squat  2 sets × 3-5 reps 24 kg  Wall facing quadruped handstand hold  2 sets × 15 reps |
| Phase C | Jump to straight legs:  4 sets × 5 24 in, each 90 sec  Single leg box jumps  4 sets × 5 + 5 20 in, each 90 sec  3 rounds:  10 jumping lunges with dumbbell (appropriate weight)  20 jumping lunges with bodyweight |
| Phase D | Hip power snatch  5 sets × 3 reps 40-50% RM, each 90 sec  15 min every minute on the minute:  1’-5’: 3 reps 70-75% RM  6’-10’: 2 reps 75-80% RM  11-15’: 1 rep 80-85% RM |
| Phase E | For time:  30 snatches 61 kg  30 bar-facing burpees  30 clean and jerks  30 bar-facing burpees 61 kg |
| **Session 2** | |
| Phase A | For time:  30 ring muscle up |
| Phase B | 4 rounds:  10 ring support swings + 10 dip swings + 10 rings support swings  20 ring swings  Rest as needed between rounds |
| Phase C | Assault bike  10 min RPE 4-5  into 8 sets:  15 sec RPE 8-9 (standing)  2 min RPE 3-4 (seated)  10 min RPE 4-5 |
